# First international workshop on human endogenous retroviruses and diseases, HERVs & disease 2015

**DOI:** 10.1186/s13100-015-0051-7

**Published:** 2015-10-15

**Authors:** Avindra Nath, Patrick Küry, Guilherme Sciascia do Olival, Antonina Dolei, Håkan Karlsson, Laurent Groc, Marion Schneider, John Kriesel, Jean-Louis Touraine, François Mallet, Patrice N. Marche, Frederick Arnaud, Cédric Feschotte, Hervé Perron

**Affiliations:** Section of infections of the Nervous System, National Institute of Neurological Diseases and Stroke, National Institutes of Health, Bethesda, MD USA; Department of Neurology, Heinrich Heine Universität Düsseldorf, Düsseldorf, Germany; Guilherme Sciascia do Olival, Emilio Ribas Infectology Institute Neuroscience and CATEM - Multiple Sclerosis Center of Santa Casa of São Paulo, São Paulo, SP Brazil; Department of Biomedical Sciences, University of Sassari, Sassari, Italy; Department of Neuroscience, Karolinska Institutet, Stockholm, Sweden; Institut interdisciplinaire de Neurosciences CNRS UMR 5297, Université Bordeaux 2, Bordeaux, France; Department of Anesthesiology, University Hospital, Ulm, Germany; Division of Infectious Diseases, University of Utah School of Medicine, Salt Lake City, UT USA; Joint Unit Hospices Civils de Lyon, bioMérieux, Cancer Biomarkers Research Group, Centre Hospitalier Lyon Sud, Pierre Bénite cedex, France; Université Lyon 1, School of Medicine, Lyon, France; INSERM U823, Université Grenoble, Grenoble, France; UMR754, Université Claude Bernard Lyon 1, Institut National de la Recherche Agronomique; Ecole Pratique des Hautes Etudes; SFR BioSciences Gerland, Lyon, France; Department of Human Genetics, University of Utah School of Medicine, Salt Lake City, UT USA; Geneuro, Geneva-Switzerland and Geneuro-Innovation, Lyon, France

## Abstract

The First International Scientific Conference on Human Endogenous Retroviruses (HERVs) and Disease, Lyon-France, May 26-27^th^ 2015, brought together scientific and medical specialists from around the world investigating the involvement of human endogenous retroviruses (HERVs) in complex human diseases.

## Introduction

The objective of this meeting was to increase awareness on emerging perspectives in this research area, which remains vibrant despite much debate and controversy over the past few decades. This meeting afforded a first opportunity to focus on the potential impact of these elements on pathological pathways leading to chronic, multifactorial and still incurable human diseases. The first steps toward innovative therapeutic developments arising from dedicated research on HERVs were also presented.

Attempts to identify among environmental viruses, a causative agent of autoimmunity, of chronic inflammation and of specific tissue lesions involving either immunological and physiological impairments as in Multiple Sclerosis (MS) or type 1 Diabetes (T1D), or neurodevelopmental and neurobiological abnormalities as in Schizophrenia (SZ) or bipolar disorder (BD), or particular neurodegenerative processes as in amyotrophic lateral sclerosis (ALS), have consistently failed. Such diseases are now considered to have a multifactorial etiology leading to complex pathophysiology and to chronic-relapsing or progressive evolution. They are most often thought to result from gene-environment interactions, in particular host susceptibility genes with infectious agents. Human genome studies have investigated DNA sequences in exons encoding somatic proteins or their associated regulatory sequences and, apart from few monogenic and inherited diseases, have generated a global vision of variable panels consisting in multiple susceptibility genes thought to cause a disease when associated. Environmental infections likely act as co-factors or triggers for the etiopathogenesis of the disease, but not as direct causative agents as in classical infectious diseases.

Since the 1980’s, a new horizon in the search for pathogenic players in complex human disease arose from studies in human cells obtained from patients with cancer or with chronic neuroinflammatory disease such as MS, in which human endogenous retrovirus (HERV) multicopy families were identified from elements expressed in association with reverse-transcriptase activity and/or virion-like particles. These observations were initially regarded as anecdotal but, as studies further explored and progressively unveiled unexpected genetic and biological features of these HERVs in association with different diseases during the past decades, it became apparent that HERVs (as other mobile genetic elements) were not merely fossils of past infection and inert relics of ancient genomic invasions, but could display activities with a substantial impact on cellular function, in both health and disease (References of review articles available from corresponding author).

Endogenous retroviruses and their relatives are currently known to occupy approximately 8 % of the human genome, are separated into diverse multicopy families and are also part of the broader category of “mobile genetic elements”, which together account for at least half of the human genome. Cumulated results from numerous studies on HERVs and related elements over the past 30 years, the number of which increased exponentially since the early years of the 21st century, brought converging and consistent observations in different disciplines as in different complex diseases. They now provide a global picture suggesting that environmental infectious agents may play a role in triggering the expression of certain HERVs with selective tropism for permissive cell phenotypes cells, which in turn may express products (RNA or protein) or cause genetic modifications, thereby engaging pathogenic pathways leading to final pathognomonic features of certain complex diseases.

These scientific advances along with the first therapeutic developments targeting HERV-W envelope protein in MS and, as now envisaged, HERV-K in ALS, are now providing a new perspective of the “gene-environment interplay” underlying the etiopathogenesis of certain complex human diseases. This prompted different groups and organizations involved in research and/or development in this novel but rapidly emerging domain to instigate an international conference gathering both basic and clinical scientists from various disciplines (genetics/genomics, immunology, virology, cell biology, etc.…) in order to share and debate current and future research on “HERV and Disease” within a multidisciplinary and transversal approach.

This international workshop on Human Endogenous Retroviruses and Diseases, “HERV & Disease”, was held 26–27th May 2015 in Lyon, France. The organizing committee consisted in: Hervé Perron (*Geneuro-Innovation*), Patrice Marche (*INSERM*), François Curtin (*Geneuro*) and Jean-Louis Touraine (*University of Lyon*). The workshop drew over a hundred attendees who actively participated in scientific exchanges and debates. Invited presentations ranged from the general biology of HERVs in the context of human genome evolution to the role of endogenous retroviruses in animal disease. The workshop included 5 scientific sessions: Neurological diseases, Virus interplay with HERV pathogeny, Neuropsychiatric diseases, Diabetes and Cancer. Here, we present highlights from the talks from all sessions.

Of note, a considerable fraction of the data presented at the workshop and reported in this article (with the consent of the authors) remains, to this date, unpublished and should be considered and treated as such and therefore not cited in other publications. Published scientific and clinical studies or reviews could not be cited in this report but references can be obtained from authors.

## Keynote addresses

Cédric Feschotte (Department of Human Genetics, University of Utah School of Medicine, Salt Lake City, UT, USA) opened the meeting with a keynote address entitled: “Of Good and Evil: Biological Impact of Endogenous Retroviruses and Other Invasive Genetic Elements”. He explained how the genomes of various eukaryotes are replete with viral sequences that are integrated or 'endogenized' in chromosomes and how recent studies have revealed that virtually all types of viruses can be endogenized. He emphasized that retroviruses are by far the best-characterized and most common source of endogenous viral elements in mammalian genomes, accounting for 6-14 % of mammalian genome thus far sequenced, and that their pervasive infiltration is a substantial source of genetic variation across and within species. He presented an overview of several well documented instances where specific ERV have influenced, for better or worse, the evolutionary trajectory of their host species. More specifically, he presented recent evidence obtained in his laboratory that ERVs have been co-opted during mammalian evolution to rewire a transcriptional network orchestrating the interferon response, a crucial arm of innate immunity. These data shed new light on the mechanisms by which misregulation of ERVs may promote disease states, such as autoimmunity and tumorigenesis.

Frédérick Arnaud (UMR754, Université Claude Bernard Lyon 1/Institut National de la Recherche Agronomique/Ecole Pratique des Hautes Etudes, Lyon, France) opened the second day with a keynote address entitled: “Friendly viruses: antiviral activities of ERVs against the oncogenic Jaagsiekte sheep retrovirus”.

He presented an original mechanism by which certain animal ERVs could confer protection to their host against a related pathogenic retrovirus, here Jaagsiekte sheep retrovirus(JSRV). JSRV is the causative agent of ovine pulmonary adenocarcinoma and coexists with related endogenous retroviruses (enJSRVs) that colonized the sheep genome throughout evolution. He explained how collaborative studies with Barbara Viginier and Christophe Terzian’s group in Lyon (University of Lyon), as well as with Alessia Armezzani and Massimo Palmarini’s group (MRC Centre for Virus Research University of Glasgow; Glasgow, Scotland) led to discover that two enJSRV proviruses (enJS56A1 and enJSRV-20), which separately entered the host genome within the last 3 million years, acquired a defective Gag polyprotein resulting in a transdominant phenotype able to block late replication steps of related JSRV. He showed how these two “protective” proviruses became fixed and amplified in the genome of domestic sheep supporting the idea of their positive selection during, or immediately before, sheep domestication (9,000 years ago). He further presented how they also identified a recent provirus (<200 years old) with an intact genomic organization that escapes the restriction induced by enJS56A1 and enJSRV-20. He concluded that the invasion of the sheep genome by endogenous retroviruses is still ongoing and has not reached an equilibrium yet. He made the audience aware of how sheep thereby provide an exciting model to study the co-evolution between ERVs and their host and to understand how ERV copies may confer resistance to a disease by interfering with related pathogenic retroviruses.

The first session, which represented most advanced studies and developments in the field of diseases associated with HERVs, took all day on May 26^th^, after welcome and introductory talks by Jean-Louis Touraine and Hervé Perron followed by the keynote lecture by Cédric Feschotte.

## Session 1: Neurological diseases and HERVs

Avi Nath (Section of infections of the Nervous System, National Institute of Neurological Diseases and Stroke, National Institutes of Health, Bethesda, Maryland, USA) reviewed consistent and independent studies that have shown an activation and upregulation of HERV-K in patients with amyotrophic lateral sclerosis (ALS). ALS is a devastating neurodegenerative disease for which there is no treatment available. Genetic mutations have been identified in nearly 20 % patients but the remaining are sporadic cases. He presented the work from his laboratory showing that all major transcripts of HERV-K were expressed in the frontal cortex of nearly 50 % patients with ALS at autopsy. He showed confirmation by quantitative polymerase chain reaction for the pol, gag and env genes and by immunostaining with an antibody to the env protein, of specific expression in cortical neurons and anterior horn cells of the spinal cord in patients with ALS but not in controls. His data from *in vitro* and *in vivo* studies indicated that HERV-K envelope protein displayed neurotoxicity for motor neurons, suggesting a pathogenic role in ALS. He concluded on a therapeutic rationale for targeting HERV-K in ALS.

Hervé Perron reminded how the first observation of retrovirus-like particles with reverse transcriptase activity was made in leptomeningeal cells shed in the cerebrospinal fluid of a patient with progressive MS in the late 1980’s. Similar observations in macrophage cultures versus controls and in EBV-transformed MS B-cells by the same and another independent group were simultaneously published in The Lancet (1991). He explained how the molecular identification of this “MS-associated” retrovirus (MSRV) RNA was achieved from purified virions in collaboration with Jeremy Garson (UCL, London, GB), as published in the Proceedings of the Academy of Sciences of the USA (1997), how sequences of a complete retroviral genome were further obtained by PCR extension with this material and, using MSRV-derived probes, how they unravelled the HERV-W family. He outlined that this novel human retrovirus did not correspond to an HTLV-like retrovirus, as suggested by Hilary Koprowski, Robert Gallo and colleagues in a study published in Nature (1985), but to HERVs that were still considered as “junk DNA”. He presented how following studies addressed the question whether this expression was a trivial activation of harmless HERV elements or whether this could be involved in MS pathogenesis. HP described the different independent confirmations that have now depicted a central pathogenic role of HERV-W envelope protein (MSRV-Env or HERV-W/Env) in the initiation and lifelong holding of MS pathogenesis. He also summarized parallel studies that showed how HERV-W activation and its envelope protein suggested a common pathogenic pathway in several other neuroinflammatory or autoimmune diseases. He gave an overview of the key aspects that were elucidated and allowed to bridge this original discovery to the clinical relevance and to an innovative therapeutic development as presented by other speakers during this session. HP concluded that, taken altogether, the present data now suggest that HERV elements could be missing links between environmental factors and complex human diseases, in which the pathogenesis has no etiological explanation. Thus, such “dormant retroviruses awaken” have been designated as “the enemy within” in recent publications and may constitute a new category of pathogens, different from classical environmental and infectious microbes, since arising from the host’s genome itself.

Patrick Küry (University of Düsseldorf, Germany) demonstrated that the envelope protein of HERV-W (MSRV-Env) affects differentiation of oligodendroglial precursor cells (OPCs), which normally remyelinate central nervous system lesions. He showed that macrophages/microglia in MS brain lesions express HERV-W/Env on the cell surface and can be found in close proximity to OPCs. Presented data showed that either recombinant protein or cells expressing HERV-W/Env prevented OPC maturation into oligodendrocytes and production of myelin proteins. This was shown to be mediated by MSRV-Env interaction with TLR4 and to induce nitrosative stress in OPCs. PK argued that data supported a direct role of HERV-W/Env in the remyelination blockade of MS lesions. He furthermore showed that this pathogenic effect was reverted by a humanized neutralizing antibody targeting MSRV-Env, thus providing perspectives of therapeutic application restoring the remyelinating potential of OPCs in MS.

Patrice Marche (U823 INSERM-University of Grenoble, France) showed that the HERV-W envelope protein (MSRV type) has specific and wide effects on brain endothelial cells with implicit consequences on the blood–brain barrier (BBB) integrity and on interactions with immune lymphoid cells. He detailed how this is again mediated by targeted activation of TLR4 pathways and, when exposed to MSRV-Env, how human brain cells (HCMEC/D3 line) or human vascular endothelial cells (HUVEC) upregulate expression of ICAM-1 integrin leading to an increased adhesion of lymphocytes to endothelial cell, followed by transmigration of lymphocytes across the endothelial barrier. These pro-inflammatory properties were observed with MSRV type HERV-W envelope but not with its HERV-W analog, Syncytin. PM reviewed recently published data from his group supporting the hypothesis that, through TLR4 pathways, MSRV-Env affects several cell types (e.g. endothelial cells, macrophages, B lymphocytes…) contributing to the immune infiltration of the brain through BBB in the pathogenesis of MS.

Antonina Dolei (Department of Biomedical Sciences, University of Sassari, Italy) discussed why immunopathogenic phenomena leading to MS could be triggered by an environmental factor operating on a predisposing genetic background. She argued that the consistent studies involved the Epstein Barr virus (EBV), and the HERV-W family including the MSRV element, able to form extracellular virions, and Syncytin-1, the envelope product of the ERVW-1 gene from an HERV-W copy on chromosome 7q21-22 with mutated and inactive gag and pol genes preventing virion production. AD presented studies from her group with repeated observations of extracellular MSRV particles, MSRV-specific mRNA sequences in MS blood, spinal fluid (CSF) or brain samples, and MRSV load paralleling MS stages, activity and therapy outcome. She detailed a long-term follow-up study, in which early MSRV detection of in CSF predicted the worst MS progression, more than ten years in advance. She further presented *in vitro* studies of HERV-W expression in blood and brain cells exposed to EBV or to EBV glycoprotein350: *in vitro,* EBV activated the HERV-W/MSRV envelope protein; HERV-W/MSRV activation is higher in blood cells of patients with infectious mononucleosis and in healthy controls with high anti-EBNA-1 IgG titers. AD concluded from these data and from the literature on MS pathogenesis that EBV could be an initial trigger of a risk for MS and that MSRV could have a direct role of effector of neurotoxicity and immunoinflammation during MS.

Jack Van Horssen (Department of biochemistry and Molecular immunology, VU-University Amsterdam, The Netherlands) presented the results of his immunohistochemical study, which showed HERV-W Env and GAG protein distribution and cellular localization in different stages of MS lesion development in a large cohort of well-characterized MS samples. He presented how HERV-W Env and GAG proteins are abundantly present in demyelinated areas with ongoing inflammation and, at the cellular level, in infiltrated macrophages as well as in activated microglia. JvH showed a moderate HERV-W Env/GAG-immunodetection in astrocytes throughout active and chronic active lesions but, within chronic inactive lesions only in few HERV-W Env-immunoreactive astrocytes. He concluded that this extensive immunohistochemical survey revealed that both HERV-W Env and HERV-W GAG protein expression is markedly upregulated in inflammatory MS lesions and, in microglia, is associated with areas of active demyelination.

John Kriesel presented results from his group having used deep sequencing to identify viral RNA signatures in human brain specimens. He explained how they had previously used this method to detect HSV1, GBV-C, and measles virus sequence in brain tissue from deceased patients. Deep sequencing was performed on brain specimens from a cohort of patients with progressive MS, revealing increased expression of HERV domains. Sequence comparisons revealed many differences for several HERV domains. After corrections for multiple tests, 2 GAGs and one KRBA sequence were significantly enriched in MS samples compared to controls. Average HRs were calculated for each domain type – AP (protease), GAG, ENV, INT, RT, and KRAB. JK presented a dendrogram constructed from hierarchical clustering that was used to discriminate MS and controls and 16 different candidate GAG and ENV domains overexpressed in MS sample have been selected for follow up RT-qPCR. He concluded that such data demonstrate that HERV and KRAB domains are significantly overrepresented in brain tissue specimens from cooresponding series, which supports the hypothesis that expression of endogenous retroviral sequences parallels MS pathogenesis, potentially reconciling the autoimmune and infectious nature of this challenging disease.

Ranjan Dutta (Department of Neurosciences, Cleveland Clinic, Cleveland, OH, USA) presented results from a recently started collaboration with the Neurology department in Düsseldorf, Germany (David Kremer and Patrick Küry). This study focused on cellular sources of HERV-W/Env expression in primary or secondary progressive MS (PPMS, SPMS) brains. At the beginning of his presentation RD reminded the audience that a cure for MS awaits the discovery of its underlying cause, though two known factors contribute to MS: a genetic predisposition and environmental agents. While few genes associated with MS susceptibility are described, limited research directly focuses on causes such as infectious agents. MS research traditionally focused on white matter demyelination although there is significant demyelination of the cortex. Demyelination happens without significant infiltration of lymphoid cells whereas recent studies suggest that cortical demyelination may exceed white matter demyelination. RD explained that a major challenge is therefore to determine how gray matter demyelination occurs. He then showed dystrophic oligodendrocytes in affected cortical regions with three-dimensional electron microscopic (3D-EM) analysis of postmortem MS brains, in which he identified proteins and organelles accumulation, whereas large scale viral sequencing analysis identified reads matching with the human endogenous retrovirus W (HERV-W) in MS brains with myelin loss. Immunostaining of demyelinated primary progressive MS cortical brain tissue with an anti-HERV-W/ENV antibody revealed its cellular localization in perivascular cuffs, within the parenchymal extracellular space, and in a microglial subpopulation. These cells also expressed HERV-W/Env receptor (TLR4) and were in direct contact with axons. RD concluded that ENV might play a role in the proinflammatory activation of microglia, thus contributing to myelin and axonal degeneration in progressive MS– a hypothesis currently under functional evaluation in both partner laboratories.

Guilherme Sciascia do Olival (Emilio Ribas Infectology Institute Neuroscience and CATEM – Multiple Sclerosis Center of Santa Casa of São Paulo, São Paulo, SP, Brazil) presented results from a collaboration with Camila Malta Romano (Luiz Henrique da Silva Nali, Augusto Cesar Penalva de Oliveira) along with the group of Neurosciencies of Emilio Ribas Institute (São Paulo, SP, Brazil). He presented results on HERV-W expression in MS patients under different treatments and disease stage with next generation sequencing analysis of the HERV-W transcripts estimating potential active loci in MS and healthy conditions and an ongoing project ERV transcriptome analysis. GSdO presented preliminary data using HERV-W envelope amplicon deep sequencing methodology on blood samples collected from 4 MS patients in relapse, from 6 MS patients with different treatments (Natalizumab and immunossupressors) and from 5 healthy subjects. He explained that the most represented transcripts in MS matched with chromosomes 1, 6, 9, 14 and X. Transcripts from healthy subjects, with lower expression, matched with chromosomes 1, 6, 7 and 14. He presented results from MS in relapse that showed elevated transcription matching with about 16 known loci but that healthy individuals presented only detectable expression matching with nine loci (p = 0.01). GSdO noticed no difference between transcription levels of patients undergoing treatments with Natalizumab and β-Interferon, compared to untreated MS cases in relapse. Interestingly, patients with high EDSS (>6) had much wider transcript sequence diversity, which consequently “matched” many more loci than patients with low EDSS (<3). He debated that this sequence diversity cannot correspond to known genomic loci, which would be in line with reported increase of HERV-W DNA copy numbers in PBMC from such patients with elevated EDSS and/or disease activity in studies by Garcia-Montojo et al. (Madrid, Spain), Perron et al. (Geneva, Switzerland) and Mameli et al. (Sassari, Italy). He concluded that HERV-W envelope expression is different between MS patients and healthy controls but that patients with high EDSS or those in relapsing conditions have more heterogeneous expression, possibly also underlying relationship between HERV-W and autoimmunity in MS.

Alain Créange (Université Paris 12 and Groupe Hospitalier Henri Mondor, Créteil, France) presented the association between chronic inflammatory demyelinating polyneuropathy (CIDP), an acquired immune-mediated inflammatory disorder of the peripheral nervous system (PNS), and HERV-W/MSRV envelope protein expression. He presented a collaborative study with the Lausanne University Hospital (CHUV-Switzerland) and GeNeuro. Env antigenaemia in CIDP patients was detected in 24/49 cases (49 %) whereas 3/18 (17 %) healthy controls were positive, which reached statistical significance (p < 0.01). Low levels of Env positivity (4/20) were observed with other neurological diseases (e.g., spastic paraparesis, metabolic neuropathies and Parkinson's disease). qRT-PCR results on peripheral blood mononuclear cells as well those of immunostaining on nerve biopsies corroborated these data. Since CIDP shares common features with MS pathogenesis, this strongly suggests that similar pro-inflammatory processes are elicited by HERV-W envelope in peripheral nerve. Such a possible involvement in CIDP might open novel perspectives for the development of targeted therapeutic approaches.

Raphaël Faucard (Geneuro-Innovation, France), presented data about Env mediated TLR4 signaling and peripheral nerve myelinating glial cells - Schwann cells - upon exposure to HERV-W Env protein. A pharmacological characterization of Env and GNbAC1, the neutralizing therapeutic antibody specifically targeting Env, was carried out using human (h)TLR4 overexpression by HEK-Blue^TM^ cells. This analysis confirmed that a) Env is a highly potent full agonist of recombinant hTLR4, b) its effects are inhibited by GNbAC1 in a concentration dependent manner and c) Env effect is mediated by its direct binding to hTLR4. It was then reported that two highly selective TLR4 inhibitors have been identified: LPS-RS as competitive ENV antagonist and Cli-095 as another signaling antagonist. RF thus argued that this was the first complete pharmacological profiling of a biological response associated to an HERV element. He further presented effects on Schwann cells exposed to Env, either transfected with or stimulated by MSRV-Env, which produced high levels of interleukin-6 (IL-6) and of chemokine CXCL10, two key immune mediators involved in CIDP pathology. He showed that effects correlated glial TLR4 expression and were inhibited by GNbAC1. He concluded that the HERV-W impact in PNS pathologies might exceed inflammation and could also include crosstalk between immune- and Schwann cells.

Alexander Emmer (Neurology department, University Hospital, Halle, Germany) then presented a recently published concept on the role and functionality of superantigens. His group previously demonstrated that T cell superantigens such as Staphylococcal enterotoxin A (SEA) can induce inflammatory reactions in different organs such as CNS, joints and muscles of experimental animals. He explained that inflammatory processes induced by the same superantigen (SEA) differed between the affected organs but mimicked corresponding human autoimmune diseases. Superantigen dependent induction of inflammation revealed independent from adjuvant mediated bypass of immune tolerance. AM showed that many autoimmune diseases display oligoclonal immunoglobulin bands in cerebrospinal fluid or different types of serum autoantibodies in other autoimmune diseases. He showed that B-cell superantigens such as gp120 (part of the HIV-envelope) can activate blood lymphocytes *in vitro* to produce immunoglobulin in an oligoclonal manner. Finally, he mentioned that another prominent feature of autoimmune diseases is organ degeneration. In many instances organ degeneration is only attributed to inflammation, however, it is important to state that degeneration may also develop in the absence of apparent major inflammation, as seen in progressive MS. He concluded that that HERV-W pathological effects may also occur in the absence of inflammation, indicating that HERV-W mode of action may also be independent of its superantigenic power.

Finally, François Curtin, the CEO of the GeNeuro gave an update on the clinical development of HERV-W Env neutralizing antibodies; following the identification of precursor murine and chimeric antibody versions, GNbAC1, a humanized IgG4 mAb, emerged from preclinical studies aiming at blocking HERV-W Env protein in MS patients. In an initial phase I trial GNbAC1 was well tolerated and only minor or nonspecific adverse events (AEs) occurred. A dose-linear pharmacokinetic profile was revealed and no anti-GNbAC1 antibodies were detected in any subject over the entire observation period of 64 days. He presented the follow up phase IIa randomised clinical study over one year, with ten MS patients randomized into two cohorts receiving an initial intravenous infusion of GNbAC1/placebo at doses of 2 or 6 mg/kg, followed by five infusions of GNbAC1 at 2 or 6 mg/kg at four-week intervals. He reported that all patients completed the 6-Months study, which was extended to one year with same regimen. Again, GNbAC1 pharmacokinetics were dose-linear, no anti-GNbAC1 antibodies were detected and no evidence of adverse effects was observed. FC highlighted that HERV-W transcripts decreased during the treatment and that nine patients had stable brain lesions at MRI (one dropped before final MRI analysis). He concluded that GNbAC1 could be a safe long-term treatment for MS patients, with promising hypothesis to be challenged in the upcoming phase IIb clinical trial.

Introducing the next sessions, which took place during the second day, Frédérick Arnaud (Lyon, Fr. and Glasgow, UK) highlighted the complex interplay between the Jaagsiekte sheep retrovirus (JSRV), causative agent of ovine pulmonary adenocarcinoma, and the highly related endogenous retroviruses (enJSRVs). These enJSRV copies not only play a critical role in conceptus development and placental morphogenesis of sheep, but can also block JSRV replication at early and late stages of the retroviral cycle (Cf. Keynote presentations).

## Session 2: Virus interplay with HERV pathogeny

The group of Elena Uleri (Department of Biomedical Sciences, University of Sassari, It.) showed that HIV tat encoded protein activates HERV-W/MSRV/Syncytin-1 in cells from blood and brain through Tat interaction with TLR4 and induction of TNF-α. *In vivo* translation suggest that Tat may promote neuroinvasion by HIV-infected macrophages, eliciting neuroinflammation and neuropathogenicity related to HERV-W elements. It was concluded that HERV-W neuropathogenic potential could contribute to NeuroAIDS pathogenesis.

Benjamin Charvet (International Centre for Infectiology Research, INSERM U1111/CNRS UMR5308/Ecole Normale Supérieure de Lyon/University of Lyon, France) analyzed the interactions between the HHV-6A herpesvirus and HERV-W, showing specific HERV-W env over-expression in HHV-6A infected glial cells, and confirming previous data of HHV-6A-induced over-expression of HERV-K18 env in lymphoid cells. He showed that HERV-W env does not involve the CD46 receptor for HHV-6A and that inactivated HHV6 virus is not efficient; whereas infectious HHV-6A induced HERV-W env transactivation in glial cells. He concluded that, when occurring, an early activation of HERV-W by HHV-6A may contribute to initiate neuroinflammatory and cytopathic effects of further self-sustained HERV-W envelope expression, thereby contributing to MS etiology.

## Session 3: Neuropsychiatric diseases and HERVs

Håkan Karlsson (Karolinska Institutet, Stockholm, Sweden) reported the identification of transcripts from HERV-W in samples from patients with schizophrenia using PCR and next-generation sequencing. He also showed transcriptional de-repression of HERV-W loci by virus infection in human cell-lines. He discussed the potential role of HERV-W as a marker or mediator of environmental risk factors in schizophrenia.

Laurent Groc (CNRS - University of Bordeaux, France) discussed the potential impact of HERV-W envelope compared to other relevant agonists, on the surface dynamics of NMDA receptors. He explained that the regulation of NMDA receptor signaling is highly dependent on the surface diffusion of the receptor and its dynamic anchoring within postsynaptic densities. He presented demonstration of a potent link between exposure to HERV-W/Env and NMDAR surface dynamics. LG emphasized that the pattern of the response was unique to HERV-W/Env compared to all other tested compounds, including LPS another TLR4 agonist. He concluded discussing the potential impact of HERV-W env expression, beyond its pro-inflammatory effects, on neuroreceptor dysregulation as observed in psychoses such as schizophrenia and bipolar disorder.

Pierre Ellul (University of Créteil, Fondation fondaMental and APHP, France) reviewed the literature on HERV expression in schizophrenia and other psychoses and hypothesized that environmental exposure to infectious or pro-inflammatory events during pregnancy cause disturbances in the epigenetic control of HERV and other retroelements which predispose to excessive transcription following secondary exposures during postnatal life. Such activation was hypothesized to contribute to the pathogenesis of these disorders.

Emanuela Balestrieri (University of Rome, Italy) disused the possible involvement of HERVs in autism. She showed distinctive expression profiles of HERV families in samples from autistic patients and a positive correlation with the severity of the clinical signs of the disease. EB further presented an animal model, consisting in outbred mice prenatally exposed to an inhibitor of the histone deacetylases (valproic acid), in which the offspring of treated mice a dysregulation in the expression of several different murine retroelements along with behavioural abnormalities, resembling those observed in individuals with autism.

## Session 4: Diabetes and HERVs

Marion Schneider (University of Ulm, Germany) recalled that type 2 diabetes (T2DM) now affects more than 387 million people worldwide with a global prevalence of 8.3 % and an estimation of about 50 % undiagnosed cases. T2DM is regarded as an important co-morbidity in depression or certain psychotic illnesses and is also one of the major risk factors for patients with major trauma to proceed into severe sepsis and septic shock in an intensive care environment. She thereafter presented her studies on patients with T2DM with sepsis focusing on peripheral blood phenotyping and highlighted the increase of non-classically activated macrophages (CD14+/C16+) in T2DM patients. MS reported how these activated cells can be enriched *in vitro* and phenotypically characterized. She explained that they consist in Immature dendritic cells (CD14-/CD16+), resembling slanDCs and M1 (STAT-1 activated), M2a-M2d macrophages and that cells from sepsis patients with and without T2DM were studied in her group. She showed that relative amounts of M1 and M2a macrophages and also slanDCs with active P2X7 ion channel activities were higher in T2DM septic patients, compared to non-diabetic patients with sepsis. She showed that cultures from T2DM patients significantly expressed s HERV-W env positive cells with simultaneous HLA-DR downregulation. She also reported that the ATP-sensitive, inflammasome-related calcium channel, P2X7 appears to be crucial for HERV-W Env positive microparticle release. The physiopathological significance of this increased occurrence of non-classically activated macrophages and of immature DC (CD14^+^/C16^+^; CD14-/CD16+) in T2DM along with the biological consequence of associated HERV-W envelope-positive microparticles release, which may eventually fuse with target cells in organs and tissue, was discussed. She concluded that both Type 1 Diabetes and T2DM with sepsis show an important link of NF-κB and STAT1 activation promoting iDC and M1 macrophage polarization, resulting in cytotoxic and inflammatory functions that may also promote HERV-W env protein expression.

Julie Medina (Geneuro-Innovation, Lyon, France) recalled that type 1 diabetes (T1D) is a severe autoimmune disease characterized by a depletion of insulin-producing beta-cells in the pancreas, that subjects with T1D are usually dependent on insulin injections for their entire life and that T1D accounts for 5 to 10 % of total number of diabetes cases, Europe having the highest incidence, with peak rates in Finland and Sardinia. She explained that both genetic factors and environmental triggers, and viruses in particular, are supposed to contribute to its etiopathogeny. After which, she explained how a study on type 1 diabetes (T1D) arose from MS preclinical study, in which serum samples from other autoimmune diseases were tested for the presence MSRV-Env antigen: no significant detection was found in, e.g., patients with Systemic Lupus (SLE) or Rhumatoid Arthritis, but was positive in sera of about 40 % of T1D with variable disease duration. JM explained how evaluating an eventual involvement of HERV-W in the pathogeny of T1D, firstly focused on HERV-W/Env expression in human pancreas biopsies from patients with and without T1D provided by the nPOD biobank (Univeristy of Florida, USA) using immunohistochemistry (IHC). She presented results showing the presence of HERV-W/Env in T1D biopsies, mainly appearing to be produced by acinar cells surrounding Langerhans islets. She further presented i*n vitro* effects of HERV-W/Env on insulin secretion when tested on cultured insulinoma cell line, which induced a progressive decrease in insulin secretion. In presence of the neutralizing antibody specific for HERV-W Env protein a normal response to glucose stimulation was restored at any of the HERV-W Env concentrations studied. Finally, she presented results showing i*n vivo* effects of HERV-W Env in a humanized NOD-SCID mouse model, showing that HERV-W Env leads to hyperglycemia and hypoinsulinemia but not in presence the monoclonal anti-HERV-W Env antibody when injected to mice at least one day after HERV-W/Env. She concluded on the present consistency between early results from human pancreatic IHC, *in vitro* and *in vitro* models, suggesting that HERV-W could be involved in T1D pathogeny. She nonetheless commented that these preliminary results call for further investigation on larger cohorts that, if in line with present observations, may provide perspectives for novel therapeutic avenues in T1D.

## Session 5: Cancer and HERVs

François Mallet (Joint Unit Hospices Civils de Lyon-bioMérieux, Lyon, France) illustrated, using high density HERV custom microarrays in Affymetrix format and a panel of 40 normal and tumor epithelial tissue pairs, that groups of transcriptionally active elements of the HERVome are sensitive to the state of cell differentiation and follow stringent tropism rules. He exemplified such tissue specificity with results of qRT-PCR follow-up of five HERV-H elements, initially identified with the HERV microarray in colon cancer, in a larger cohort of 212 patients; samples comprised adenomas, colorectal carcinomas and liver metastasis, normal and tumoral liver tissue, as well as organs with common sites of metastasis. FM showed that HERV-H expression was maintained throughout disease progression and strongly correlated with microsatellite instability in colon cancer.

Reiner Strick (University Clinic Erlangen, Erlangen, Germany) proposed a candidate/functional gene approach to decipher HERV expression by immunohistochemistry or q-PCR in 135 brain tumors consisting of pituitary adenomas (PA) subtypes as well as of glioblastoma multiforme (GBM) and giant cell GBM as compared to normal adenohypophysis and cortex. He presented Syncytin-1 and 20 ERV functional envelopes expression study and reported highly up-regulated Syncytin-1, Syncytin-2, erv-3 and envK in different PA subtypes, GBM and giant cell GBM. RS concluded that HERV genes play an essential role in PA and GBM, possibly via general cAMP signaling, where Syncytin-1 could be one main factor contributing to the multinucleated cells in giant cell GBM.

Claudia Matteucci (University of Roma, Roma, Italy) presented results on the potential role of HERV-K activation in cellular plasticity and stemness features of melanoma cells upon the modification of the microenvironment, recalling that her group previously demonstrated that HERV-K was associated to aggressiveness and immune evasion of metastatic melanoma using stress culture conditions. CM showed that combination of flow cytometry, RT-PCR analyses, RNA interference, sphere-forming and migration/invasion assays, using the highly heterogenic human melanoma cell line TVM-A12 and other commercial cell lines, confirmed that melanoma cell plasticity and generation of CD133+ putative cancer stem cell subpopulation are HERV-K dependent. She concluded on a promising observation showing that TVM-A12-CD133+ cells were affected by non-nucleoside reverse transcriptase inhibitors.

Martin Sebastian Staege (Martin Luther University, Halle-Wittenberg, Germany) presented his studies on HERV expression in Hodgkin’s lymphoma (HL) cells, combining conventional DNA microarray analysis for the characterization of gene expression in HL cells compared to normal blood cells with *in silico* identification of HERV located in the vicinity of expressed genes and with wet lab experiments on switching mechanism at 5' end of RNA transcript to validate new HERV-defined alternative transcription start sites. He concluded that data obtained by his group indicated that HERV can recruit alternative promoters in HL cells.

## Concluding remarks

This first iteration of the ‘HERV & Disease’ workshop provided the opportunity to bring together scientists and clinicians with diverse expertise to share data and ideas about the biology of HERVs and the possible impact of these elements in health and disease. The event came more than two decades after the discovery of the first HERV-W element isolated from an MS patient, a finding that has been the subject of much controversy and debate in the community. How could viral elements assimilated within the genome of humans trigger diseases in certain patients but not others? Many laboratories have invested a considerable effort to clarify this issue, yielding important clues acting at different levels. First, recent advances in genomics have started to provide solutions to the technical challenges posed by the genetic complexity and repetitive nature of HERVs in the human genome. This is best illustrated with HERV-K insertions, a growing number of which have now been shown to be polymorphic and to occur at low frequency in the human population. Furthermore, at the gene-environment interface, there is growing recognition of cross-talks between diverse infectious agents (including non-retroviral viruses) and HERVs and of their interference with inflammatory and cytopathic signaling pathways. This particular role of environmental microbes provides examples of etiopathogenic mechanisms by which non-physiological HERV activation may occur and may have biological effects. This would confer a “hit and run” role for the many infectious factors often but partially associated with these diseases and a central role for HERVs that, once transcriptionally activated, can trigger and fuel downstream pathogenic cascades leading to specific lesions or cellular dysfunctions. In parallel, diseases associated with the activation of elements of the HERV-W family have now been extended from MS to other inflammatory neurological or neuropsychiatric diseases, at least one other autoimmune disease (T1D) and, partly or in association with other HERVs, in some cancer states. In the case of HERV-K, evidence is mounting for the involvement of the HERV-K envelope protein in the etiopathogenesis of sporadic SLA, which represents a potential breakthrough for our understanding of this and other neurodegenerative diseases. So, this and many other studies on HERVs open exciting prospects as well as new challenges to elucidate multifactorial etiopathogenic pathways and cellular mechanisms underlying the etiology and progression of many poorly understood pathologies. There is great hope that innovative therapies will emerge from this research. In this respect, the good results from early clinical trials (Phase I in healthy volunteers and Phase IIa in MS patients) for the first specific immunotherapy targeting an associated pathogenic HERV protein (references of publications available from the corresponding author), provide encouraging and new perspectives for the patients.

The initiative of such an international meeting on ‘HERV & Disease’ is expected to continue. Information about a new iteration of this workshop will be available on the website of the meeting before the end of 2015. Also note that abstracts are freely available online: www.hervanddisease.com

